# More severe hypoxemia is associated with better subjective sleep quality in obstructive sleep apnea

**DOI:** 10.1186/s12890-015-0112-1

**Published:** 2015-10-12

**Authors:** Meng-Ni Wu, Chiou-Lian Lai, Ching-Kuan Liu, Li-Min Liou, Chen-Wen Yen, Sharon Chia-Ju Chen, Cheng-Fang Hsieh, Sun-Wung Hsieh, Feng-Cheng Lin, Chung-Yao Hsu

**Affiliations:** Department of Neurology, Kaohsiung Medical University Hospital, Kaohsiung, Taiwan; Department of Master’s Program in Neurology, Faculty of Medicine, College of Medicine, Kaohsiung Medical University, No. 100 Tzyou 1st Road, Kaohsiung, 807 Taiwan; Department of Neurology, Kaohsiung Municipal Hsiao-Kang Hospital, No. 482 Shanming Rd., Siaogang Dist., Kaohsiung City, 812 Taiwan; Department of Mechanical and Electro-mechanical Engineering, National Sun Yat-Sen University, No. 70 Lienhai Rd., Kaohsiung, 80424 Taiwan; Department of Medical Imaging and Radiation Sciences, Kaohsiung Medical University, No. 100 Tzyou 1st Road, Kaohsiung, 807 Taiwan; Division of Geriatrics and Gerontology, Department of Internal Medicine, Kaohsiung Medical University Hospital, Kaohsiung, Taiwan; Graduate Institute of Medicine, College of Medicine, Kaohsiung Medical University, No. 100, Tzyou 1st Road, Kaohsiung, 807 Taiwan; Department of Health, Pingtung Hospital, No. 270, Ziyou Rd., Pingtung City, Pingtung County 900 Taiwan

**Keywords:** Compliance, Continuous positive airway pressure, Hypoxemia, Obstructive sleep apnea, Perception, Sleep quality

## Abstract

**Background:**

Perceived sleep quality may play an important role in diagnosis and therapy for obstructive sleep apnea (OSA). However, few studies have assessed factors that are associated with perceived sleep quality in OSA patients. Hypoxemia depresses the central nervous system and attenuates the perceived respiratory load in asthmatic patients. This study aimed to investigate the factors related to perceived sleep quality, focusing on the role of hypoxemia.

**Methods:**

Polysomnography studies of 156 OSA patients were reviewed. Traditional polysomnographic parameters, including parameters of oxy-hemoglobin saturation (SpO2), were calculated, and the sleep questionnaire and scales were used. Considering the possible pitfalls of absolute values of SpO2 and individualized responses to hypoxemia, the amplitude of desaturation was further computed as “median SpO2 minus lowest 5 % SpO2 “and “highest 5 % SpO2 minus median 5 % SpO2”. Correlations between these parameters and perceived sleep quality, represented as the Pittsburgh sleep quality index (PSQI), were performed. Multiple linear regression analysis was also conducted to investigate the factors associated with the PSQI.

**Results:**

Although the PSQI was not correlated with the apnea-hypopnea index (r = −0.113, *p* = 0.162) and oxygen desaturation index (r = −0.085, *p* = 0.291), the PSQI was negatively correlated with “median SpO2 minus lowest 5 % SpO2” (r = −0.161, *p* = 0.045). After adjusting for age, total sleep time, the periodic limb movements index, tendency of depression, and the lowest 5 % SpO2, the “median SpO2 minus lowest SpO2” was still a significant predictor for a lower PSQI (β = −0.357, *p* = 0.015).

**Conclusions:**

More severe hypoxemia is associated with better perceived sleep quality among OSA patients. This paradox may be associated with hypoxemia-related impairment of perception. The effect of hypoxemia did not appear to be significant in relatively mild hypoxemia but become significant in severe hypoxemia.” Median SpO2 minus lowest 5 % SpO2” may also be a better predictor of perceived sleep quality than the apnea-hypopnea index because of the disproportionate effects of hypoxemia. Additionally, further studies are necessary to confirm the role of hypoxemia on perceived sleep quality and identify the possible threshold of hypoxemia in OSA patients.

## Background

Obstructive sleep apnea (OSA) is characterized by repetitive cessation of breathing with preserved ventilatory effort during sleep. OSA is common in the general population, with a prevalence of 24 % in men and 9 % in women [1]. The prevalence of OSA has increased in the last two decades [2]. OSA has been shown to be associated with excessive daytime sleepiness, poor attention, impaired cognitive function, and increased cardiovascular risks [3, 4]. Even though undiagnosed OSA results in potential health risks and increases the socio-economic burden [5], 82–93 % of OSA remains clinically undiagnosed [6]. Because only 15.5–22.6 % of OSA patients can subjectively identify symptoms that lead them to seek medical assistance, misperception of sleep quality may play a crucial role in undiagnosed OSA [1]. Additionally, misperception of sleep quality may further reduce the motivation to have continuous positive airway pressure (CPAP) therapy. Conversely, pre-CPAP health belief-modifying interventions can improve compliance to CPAP [7–9]. Although perception of sleep quality is important in diagnosis and therapy of OSA, few studies have examined factors associated with perception of sleep quality in OSA patients.

Hypoxemia, one of the main presentations of OSA, depresses the central nervous system, and attenuates the perception of respiratory load and dyspnea in severe asthmatic patients. Hypoxemia may also delay the reaction time of the nervous system after thermal, visual, and auditory stimulation [10, 11]. In addition, hypoxemia can stimulate inhibitory neurotransmitters, such as endogenous opioids, adenosine, and gamma-aminobutyric acid. These neurotransmitters may be responsible for a depressed response of the central nervous system and attenuated perception [12, 13]. However, whether hypoxemia may also be related to attenuated perception of sleep quality in OSA patient is still unclear.

Because perception of sleep quality is subjective and difficult to quantify, a well-established and self-rated questionnaire may be an appropriate way of measurement. The Pittsburgh sleep quality index (PSQI) has been extensively used in a variety of populations. The internal homogeneity, consistency, validity, sensitivity, and specificity of the PSQI are acceptable [14]. The PSQI has been used in several psychometric studies, including in patients with bone marrow transplant, renal transplant, breast cancer, benign breast problems, and primary insomnia [15, 16]. Moreover, the PSQI is also used as a surrogate of perception of sleep quality in patients of chronic fatigue syndrome, myasthenia gravis, dementia, healthy women, and even in patients with OSA [17–21].

Therefore, this retrospective study was conducted to investigate factors that are associated with the perception of sleep quality by analyzing the PSQI, focusing on the role of hypoxemia, in OSA patients.

## Methods

### Patients

Records of patients who were referred to the Sleep Center of Kaohsiung Medical University Hospital for their first diagnosis of OSA between January 2009 and December 2011 were reviewed. This study was approved by the Institutional Review Board of Kaohsiung Medical University Hospital (KMUH-IRB-20120136). Because of the nature of retrospective design in this study, no informed consent was available.

Patients with an apnea-hypopnea index (AHI) ≥ 5 per hour and a diagnosis of OSA were enrolled. Those with other concurrent sleep diseases, including periodic limb movement disorder, restless leg syndrome, rapid eye movement behavior disorder, and narcolepsy were excluded. Patients with known medical or neurological diseases that might disturb sleep quality (i.e., congestive heart failure, asthma, chronic obstructive pulmonary disease, chronic kidney disease, liver cirrhosis, severe osteoarthritis, malignancy, dementia, Parkinsonism, or disabling stroke) were also excluded. All of the patients underwent one overnight full-channel polysomnography (PSG) and completed sleep questionnaire and scales in terms of the Chinese versions of the Epworth sleepiness scale (ESS), the PSQI, the short form-36 (SF-36), and either the Center for Epidemiologic Studies Depression Scale (CES-D) or the Hospital Anxiety and Depression Scale (HADS) before receiving PSG. This study was approved by the Institutional Review Board of Kaohsiung Medical University Hospital (KMUH-IRB-20120136).

### Polysomnography

The overnight full-channel PSG was recorded by two validated machines (Respironic Alice 5 or Nicolet Ultrasom), including six referential channels of an electroencephalogram (EEG): F3-A2, F4-A1, C3-A2, C4-A1, O1-A2, O2-A1, two channels of an electrooculogram recorded on bilateral canthi, and three channels of an electromyogram recorded on the submentalis and bilateral tibialis anterior muscles. Nasal airflow was recorded by a thermistor and a nasal airway pressure transducer. Respiratory effort was recorded by thoracic and abdominal respiratory inductive plethysmography. Oxyhemoglobin saturation (SpO_2_) was recorded by pulse oximetry and the probe was placed on the patient’s index finger.

Sleep stages and sleep-related events were scored based on the criteria of the American Academy of Sleep Medicine [22]. EEG arousal was defined as at least 3 seconds of an abrupt shift in EEG frequency that was preceded by at least 10 seconds of sleep on any of the referential EEG channels. The frequency of arousals was represented as the arousal index (AI) which was calculated by arousal events per hour of total sleep time. Apnea was defined as a drop in the peak thermal sensory excursion by more than 90 % of baseline lasting at least 10 seconds. Hypopnea was defined as a decrease in airflow either by ≥ 30 % of baseline associated with at least 4 % of desaturation, or ≥ 50 % of baseline, accompanied by arousal or ≥ 3 % of desaturation from the pre-event baseline.

The AHI was calculated by the number of apnea and hypopnea events per hour of total sleep time. The oxygen desaturation index (ODI) was calculated by the number of desaturation events ≥ 4 % compared with baseline divided by the total sleep time. Other respiratory event-related parameters, including mean saturation (mean SpO2) and minimum saturation (mini SpO2) were also analyzed. The time spent with saturation < 90 % (T90) was defined as the total duration (seconds) of desaturation with SpO2 < 90 % per hour of total sleep time.

An episode of leg movement (LM) was defined as an increase in amplitude of an electromyogram greater than 8 μV from baseline for 0.5–10 seconds. More than four consecutive LMs with a 5–90 second interval between the start of two LMs was defined as periodic limb movements in sleep (PLMS). The periodic limb movements index (PLMI) was calculated by dividing the total events of PLMS by the total sleep time (hours).

### Sleep questionnaire and scales

All of the patients completed validated sleep questionnaires and sleep scales using the Chinese versions of the ESS, PSQI, SF-36, and either the CES-D or HADS. The ESS comprises eight items, with a total score of 0–24, where a higher ESS score indicates worse daytime sleepiness [23]. The PSQI consists of nine items with a total score of 0–21 and seven sub-scores in terms of (1) subjective sleep quality, (2) sleep latency, (3)sleep duration, (4) habitual sleep efficiency, (5) sleep disturbance, (6) use of hypnotics, and (7) daytime dysfunction. A higher PSQI score indicates worse subjective sleep quality [24].

The SF-36 is a self-rated scale that is widely used for assessing health-related quality of life. The SF-36 consists of eight subscales, including physical functioning, role physical, role emotion, vitality, mental health, social functioning, body pain, and general health. Each item is scored from 0–100 and integrated into two summary measures, the physical and mental component scores of the SF-36 (SF-36 physical and SF-36 mental). Higher scores in the SF-36 indicate better health-related quality of life [25]. The CES-D, designed to evaluate the patient’s tendency of depression, contains 20 subscales, with each score ranging from 0–4. Higher scores suggest a higher possibility of depression [26].

Finally, the HADS is a self-administered 14-item scale which is used to investigate the level of anxiety and depression. The HADS is divided into the HADS-anxiety (HADS-A) and HADS-depression (HADS-D). Each part contains seven subscales with a score ranging from 0–3. Higher scores indicate a greater tendency of anxiety or depression. Depression was defined in this study if either the CES-D score was ≥ 15 or the HADS-D score was ≥ 8 [27].

### Saturation-related parameters

Signals of SpO2, sampled at 1 Hz by pulse oximetry, were analyzed by Matlab. Artifacts, defined as an abrupt change in SpO2 > 4 % compared with baseline (one second before the artifact), were excluded and replaced with SpO2 one second before the artifact [28]. SpO2 values were further rearranged from lowest to highest to establish an SpO2 series. The median value of the SpO2 series (median SpO2), the average value of the lowest 5 % of the SpO2 series (lowest 5 % SpO2), and the average value of the highest 5 % of the SpO2 series (highest 5 % SpO2) were calculated. Furthermore, the amplitude of desaturation was computed based on the difference between the highest 5 % SpO2 and the median 5 % SpO2 (highest 5 % SpO2 minus median 5 % SpO2), as well as the difference between the median SpO2 and the lowest 5 % SpO2 (median SpO2 minus lowest 5 % SpO2).

### Statistical analysis

Statistical analysis was performed by JMP version 9 (SAS Institutes Inc, Cary, NC, USA). Continuous variables (i.e., age, PSG parameters, sleep questionnaire/scales, and saturation-related parameters) are shown as mean ± standard deviation (SD). Binary variables (sex) are shown as the number of patients and percentage. The Student’s *t*-test was used to compare the PSQI between men and women. Pearson’s correlation was used to assess the relationships among demographic variables, PSG parameters, sleep questionnaire/scales, saturation-related parameters, and the PSQI.

Mixed model stepwise multiple linear regression analysis was performed to determine factors associated with the PSQI. Candidate variables were chosen to be entered into multivariable analysis if *p* <0.2 was identified in Pearson’s correlation analysis and the Student’s *t* test. Determination of collinearity was performed by the attached software from JMP by which the variance proportion, the eigenvalue, and the condition index were available. Collinearity of these variables was decided if the condition number, defined as the maximum of the condition index, was more than 30. If collinearity was identified, centering was conducted. The entered variables were presented with their coefficient (β), 95 % confidence interval (95 % CI), and *p* value after adjusting for other confounding variables. Statistical significance was set at *p* < 0.05.

## Results

Of 325 patients, 247 who fulfilled the inclusion criteria were enrolled. Based on a chart review, 91 patients with other concurrent sleep disorders or known medical or neurological disease were excluded. The remaining 156 patients were included in the final analysis.

### Baseline data

Table 1 shows the baseline demographic characteristics of all OSA patients. The 156 OSA patients included 118 (75.6 %) men and 38 (24.4 %) women. The mean (±SD) age was 47.3 ± 14.0 years. The mean (±SD) of AHI, ODI, mean SpO2, mini SpO2, and T90 were 35.0 ± 25.9/h, 30.7 ± 24.7/ h, 94.7 % ± 2.4 %, 77.5 ± 10.4 %, and 445.6 ± 571.5 s/h, respectively. The mean (±SD) of total sleep time, sleep efficiency, and AI were 355.0 ± 61.8 minutes, 83.2 ± 12.6 %, and 31.8 ± 23.4/h, respectively. The mean (±SD) PSQI was 9.4 ± 4.3, and the mean (±SD) ESS was 11.2 ± 5.3. According to either the CES-D or the HADS-D, 46.8 % of patients had a tendency for depression.

**Table 1:** Demographic characteristics of OSA patients

Characteristics, (*n* = 156)	Mean (SD)
Gender-male (n, %)	118 (75.6 %)
Age(years)	47.3(14.0)
BMI (Kg/m^2^)	27.3 (4.5)
Total sleep time (min)	355.0(61.8)
Sleep efficiency(%)	83.2(12.6)
Sleep latency(min)	14.3(19.7)
Stage I (%)	23.8(19.4)
Stage II (%)	51.3(13.5)
Stage III (%)	11.3(10.4)
Stage REM (%)	13.5(6.7)
AHI (/hour)	35.0(25.9)
Mean SpO2 (%)	94.7(2.4)
Mini SpO2 (%)	77.5(10.4)
ODI (/hour)	30.7(24.7)
T90 (second/hour)	445.6(571.5)
Arousal index (/hour)	31.8(23.4)
PLMI (/hour)	0.4(0.8)
PSQI (scale)	9.4(4.3)
ESS (scale)	11.2(5.3)
SF-36 physical (scale)	46.8(9.2)
SF-36 mental (scale)	41.3(11.6)
CESD (*n* = 36)	18.0(11.0)
HADS-depression(*n* = 120)	6.5(3.8)
Median SpO2 (%)	94.9(2.6)
Lowest 5 % SpO2 (%)	81.4(9.4)
Highest 5 % SpO2 (%)	98.2(0.9)
Hightest 5 % SpO2 minus Median SpO2	3.28(2.28)
Median SpO2 minus Lowest 5 % SpO2 (%)	13.5(8.3)

*Abbreviations*: *BMI* body mass index; *REM* rapid eye movement sleep; *AHI* apnea-hypopnea index; *Mean SpO2* mean saturation of total time in bed; *Mini SpO2* minimum saturation of total time in bed; *ODI* oxygen desaturation index; *T90* time spent with saturation < 90 %; *PLMI* periodic leg movement index; *PSQI* Pittsburgh sleep quality index; *ESS* Epworth sleepiness scale; *SF-36 Physical*, physical component score of short form 36; *SF-36 Mental*, mental component score of short form 36; *CESD* center for epidemiologic studies depression scale; *HADS-depression* depression component of Hospital Anxiety and Depression Scale; *median SpO2* the median value of saturation of total sleep time; *Lowest 5 % SpO2* averaged value from the lowest 5 % saturation of total sleep time; *Highest 5 % SpO2*, averaged value from the highest 5 % saturation of total sleep time; *Highest% SpO2 minus Medium SpO2*, the difference between Highest 5 % SpO2 and Medium SpO2; *Median SpO2 minus* Lowest 5 % SpO2, the difference between Median SpO2 and Lowest 5 % SpO2

### Univariate analysis

The PSQI was not significantly different between men and women (mean:9.1; 95 % CI :8.36–9.85 vs. mean:10.32; 95 % CI:8.73–11.90, *p* = 0.129). Table 2 shows the relationship between the PSQI and age, body mass index, traditional PSG parameters, sleep questionnaire and scales, and saturation-related variables. There were significantly positive correlations between the PSQI and age (r = 0.209, *p* = 0.009), ESS (r = 0.274, *p* < 0.001), CES-D (r = 0.372, *p* = 0.026), and HADS-D (r = 0.257, *p* = 0.005).

**Table 2:** Correlation between PSQI-total score and age, body mass index, traditional parameters of polysomnography, sleep questionnaire and scales, and saturation-related variables (*n* = 156)

Variables	Correlation	Lower 95 %	Upper 95 %	*p*-value
Demographic data				
Age(years)	0.209	0.054	0.355	0.009*
BMI (Kg/m^2^)	0.021	−0.137	0.177	0.796
Parameters of PSG				
Total sleep time (min)	−0.235	−0.379	−0.081	0.003*
Sleep efficiency(%)	−0.195	−0.342	−0.039	0.015*
Sleep latency(min)	0.114	−0.044	0.267	0.156
Stage I (%)	0.050	−0.108	0.206	0.533
Stage II (%)	0.001	−0.156	0.158	0.992
Stage III (%)	−0.077	−0.231	0.081	0.339
Stage REM (%)	−0.016	−0.172	0.142	0.846
AHI (/hour)	−0.113	−0.265	0.045	0.162
Mean SpO2 (%)	0.016	−0.142	0.172	0.846
Mini SpO2 (%)	0.084	−0.075	0.238	0.300
ODI (/hour)	−0.085	−0.239	0.073	0.291
T90 (second/hour)	−0.069	−0.223	0.090	0.396
Arousal index (/hour)	−0.023	−0.179	0.135	0.778
PLMI (/hour)	0.146	−0.011	0.296	0.069
Questionnaire				
ESS	0.274	0.122	0.413	<0.001*
SF-36 physical	−0.394	−0.519	−0.253	<0.001*
SF-36 mental	−0.445	−0.563	−0.310	<0.001*
CESD (*n* = 36)	0.372	0.049	0.624	0.026*
HADS-depression (*n* = 120)	0.257	0.081	0.418	0.005*
Saturation-related parameters				
Median SpO2	−0.096	−0.249	0.062	0.235
Lowest 5 % SpO2	0.115	−0.043	0.267	0.154
Highest 5 % SpO2	−0.149	−0.299	0.008	0.063
Highest % SpO2 minus	0.051	−0.107	0.206	0.526
Median SpO2				
Median SpO2 minus Lowest 5 % SpO2	−0.161	−0.310	−0.004	0.045*

*p < 0.05

*Abbreviations*: *BMI* body mass index; *REM* rapid eye movement sleep; *AHI* apnea-hypopnea index; *Mean SpO2* mean saturation of total time in bed; *Mini SpO2* minimum saturation of total time in bed; *ODI* oxygen desaturation index; *T90* time spent with saturation < 90 %; *PLMI* periodic leg movement index; *ESS* Epworth sleepiness scale; *SF-36 Physical* physical component score of short form 36; *SF-36 Mental* mental component score of short form 36; *CESD* center for epidemiologic studies depression scale; *HADS-depression* depression component of Hospital Anxiety and Depression Scale; *median SpO2* the median value of saturation of total sleep time; *Lowest 5 % SpO2* averaged value from the lowest 5 % saturation of total sleep time; *Highest 5 % SpO2* averaged value from the highest 5 % saturation of total sleep time; *Highest% SpO2 minus Medium SpO2* the difference between Highest 5 % SpO2 and Medium SpO2; *Median SpO2 minus Lowest 5 % SpO2* the difference between Median SpO2 and Lowest 5 % SpO2

There were significantly negative correlations between the PSQI and total sleep time (r = −0.235, *p* = 0.003), sleep efficiency (r = −0.195, *p* = 0.015), and the SF-36 physical and the SF-36 mental (r = −0.394, *p* < 0.001 and r = −0.445, *p* < 0.001, respectively). However, there were no significant correlations between the PSQI and the AHI, ODI, mean SpO2, mini SpO2, or T90.

When we analyzed the relationships between the PSQI and saturation-related parameters, there were still no significant correlations between the PSQI and median SpO2, lowest 5 % SpO2, highest 5 % SpO2, and “highest 5 % SpO2 minus median SpO2”. The PSQI did correlate negatively with “median SpO2 minus lowest 5 % SpO2” (r = −0.161, *p* = 0.045).

### Multivariable analysis

By mixed model stepwise multiple linear regression analysis (Table 3), the larger“median SpO2 minus lowest 5 % SpO2” value was still significantly associated with a lower PSQI(β = −0.357; 95 % CI: −0.644– -0.070, *p* = 0.015) after adjusting for age, total sleep time, PLMI, tendency of depression, and lowest 5 % SpO2.

**Table 3:** Multiple linear regression (stepwise with mixed entry)

Term	Coefficient	SE	Lower 95 %	Upper 95 %	*p*-value
Intercept	12.866	2.000	8.913	16.818	<.0001
C-age (years)	0.053	0.023	0.007	0.099	0.025*
C-total sleep time (mins)	−0.010	0.005	−0.021	0.001	0.064
PLMI (/hr)	0.618	0.385	−0.142	1.378	0.110
Tendency of depression	2.378	0.636	1.120	3.635	<.0001*
C-lowest 5 % SpO2 (%)	−0.263	0.128	−0.516	−0.009	0.042*
Median SpO2-Lowest 5% SpO2	−0.357	0.145	−0.644	−0.070	0.015*

*p < 0.05

*Abbreviations*: *C-age* centering of age (age minus the mean of age); *C-total sleep time* centering of total sleep time (total sleep time minus the mean of total sleep time); *C-lowest 5 % SpO2* centering of the averaged value from the lowest 5 % saturation of total sleep time (lowest 5 % SpO2 minus the mean of lowest 5 % SpO2). These centering were performed since collinearity was diagnosed; *PLMI* periodic leg movements index; tendency of depression was defined as either CES-D ≥15 or HADS-D ≥ 8; *median SpO2-lowest 5 % SpO2* difference between median to lowest 5 % of saturation of total sleep time. Median SpO2 minus Lowest 5 % SpO2 was the independent predictor for lower PSQI after adjusting other variables

## Discussion

This study showed that there was no significant correlation between the PSQI total score and traditional PSG parameters, including the AHI and ODI. Nonetheless, the amplitude of desaturation in terms of “median SpO2 minus lowest 5 % SpO2” was a significant predictor for a lower PSQI total score although “highest 5 % SpO2 minus median SpO2” was not correlated with the PSQI. These findings suggest that OSA patients with more severe hypoxemia paradoxically feel that they have better sleep quality. Moreover, the effect of hypoxemia on perceived sleep quality was not significant in relatively mild hypoxemia, but appeared to become remarkable in severe hypoxemia.

Perception of sleep, a complicated process of cognition, involves several subjective experiences, including sensory and visual imagery, affect, and reality orientation. Three other types of cognition, including thought control, awareness of surroundings, and temporal awareness, are significantly correlated with subjective estimation of physiological sleep states [29]. Many areas in the brain, including the thalamus, hypothalamus, cingulate gyrus, right insula, and temporal, inferior parietal, and frontal lobes, are involved in the perception of sleep [29, 30]. Most of these areas are also involved in OSA patients, as supported by neuro-imaging studies.

High-resolution magnetic resonance imaging by voxel-based morphometry shows gray matter loss in the frontal, temporal, and parietal lobes, the anterior cingulate gyrus, the hippocampus, and the cerebellum in patients with OSA [31]. Functional magnetic resonance imaging demonstrates decreased brain activation in the frontal and parietal lobes, insula, and cingulate gyrus [32], while single photon emission computerized tomography shows significantly reduced regional cerebral blood flow in the bilateral para-hippocampal gyri, right lingual gyrus, peri-central gyrus, and cuneus in OSA patients [33]. Taken together, these findings suggest that dysfunction in these areas can account not only for cognitive deficits, but also for the misperception of sleep quality in OSA patients.

There is other evidence to support hypoxemia-related misperception. Exposure to normobaric hypoxia reduces neural activity and slows conduction velocity in the sensor-to-effector pathway of cutaneous warm and cold sensations [10]. Exposure to hypoxia delays the reaction time of visual and auditory responses in a dose-dependent effect [11]. Hypoxia also increases the threshold of sensation of breathlessness during an exercise challenge and can diminish the perception of dyspnea, as well as the magnitude of an externally applied resistive load [13]. Moreover, hypoxemia stimulates some inhibitory neurotransmitters, including endogenous opioids, adenosine, and gamma-aminobutyric acid, which further depress the central nervous system and attenuate perception [12]. Therefore, in this study, hypoxemia was assumed to play an important role in the misperception of sleep quality in patients with OSA.

With regard to the effect of hypoxemia, two major characteristics of hypoxemia were considered: time-associated factors, including frequency or duration of hypoxemia, and the severity of hypoxemia. Widely used traditional PSG parameters, including the AHI and ODI, were designed to assess the frequency of respiratory events. However, detailed information on the severity of hypoxemia was not available. Therefore, these traditional PSG parameters were assumed not to be a sensitive predictor for investigating hypoxemia-related misperception of sleep quality. The current study showed that these traditional PSG parameters were not correlated with the PSQI total score, as reported by Scarlata et al. [34].

Although SpO2 can reflect the severity of hypoxemia, some problems need to be resolved. First, there may be measurement errors of SpO2 detected from pulse oximetry. To minimize this error, detected SpO2 was replaced with data one second before it if SpO2 abruptly changed > 4 % compared with baseline. Second, the same value of detected SpO2 by pulse oximetry may not represent the same severity of insufficient oxygenation in different patients because the detected SpO2 may be affected by the patient’s skin color and peripheral circulation. Therefore, we focused on the amplitude of desaturation rather than the absolute value of SpO2 in this study to minimize the inter-rater difference of detected SpO2. Third, responses to hypoxemia vary according to the individual. Hypoxemia with a desaturation of 10 % may result in a profound effect on diseased elderly people, whereas hypoxemia with the same desaturation may only cause a mild effect on healthy young people. Considering individualized responses to hypoxemia, we calculated saturation-related parameters by percentile rank. Furthermore, we used the average value of the lowest and highest 5 % of the SpO2 series (lowest 5 % SpO2 and highest 5 % SpO2) to avoid outliers and fluctuation of SpO2.

T90, another widely used parameter, represents the duration of severe hypoxemia. However, the problems of absolute values of SpO2 and individualized responses to hypoxemia may persist. Additionally, whether SpO2 of 90 % is the most appropriate cutoff value for perceived sleep quality needs to be carefully considered because the time spent with saturation < 95 % is correlated with other physiological changes in OSA patients [35]. The current study showed that T90 was not correlated with the PSQI. We speculate that T90 may not be a sensitive predictor for misperception of sleep quality.

By analyzing the relationship between the PSQI and saturation-related parameters, our study showed that more severe hypoxemia was associated with better perceived sleep quality. However, the effect of hypoxemia on perceived sleep quality did not appear to be significant in relatively mild hypoxemia, but become significant if the hypoxemia was severe. Similar results have been reported in previous studies which showed that the effects of hypoxemia, measured either by visual or auditory reaction time, are not profound until saturation is below the threshold [11]. The correlation between hypoxemia and perceived sleep quality does not appear to be linear and a threshold of hypoxemia may exist. Therefore, saturation-related parameters, such as “median SpO2 minus lowest 5 % SpO2” may be better predictors of the effect of hypoxemia on patients with OSA.

Few studies have focused on the perception of sleep quality in OSA patients but the results remain inconclusive. Some studies have shown similar findings to the current study. Krakow et al. demonstrated that OSA patients complaining of insomnia or poor sleep quality had a lower AHI compared with those with better subjective sleep quality [36]. Krell et al. showed that patients with a higher AHI presented with less insomnia than patients without significant sleep-disordered breathing [37]. Gooneratne et al. also showed a higher AHI in patients without sleep complaints (79 %) than in those with sleep problem (62.6 %) [38]. However, in these studies, the patients were simply divided into groups with and without sleep complaints by subjective definitions [13, 36–38].

Other studies have shown contradictory findings. Smith et al. showed no correlation between severity of OSA and the perception of sleep quality, measured by either the PSQI or subjective complaints [39]. However, there were no adjustments for possible confounding factors in these studies. In contrast to previous studies, our study used the validated Chinese version of the PSQI to quantify subjective sleep quality and conducted multivariable analysis to adjust for confounding factors.

This study has some limitations. Even though PSG is a standard diagnostic tool for OSA, one night of PSG may not precisely represent the severity of OSA because of night-to-night variability of the AHI. In addition, the PSQI is designed to represent subjective sleep quality one month before filling in the scale, whereas PSG only represents data on the night of examination. Whether PSG data are correlated with PSQI scores warrants further investigation. Also, this study is retrospective in nature, and thus a control group was not available. Furthermore, comorbid medical or neurological disorders that may disturb sleep quality were excluded based on the selection criteria, which may have affected generalization of our results.

## Conclusions

This study suggests a paradox of better perceived sleep quality among OSA patients with more severe hypoxemia, and this may be associated with hypoxemia-related impairment of perception. The effect of hypoxemia does not appear to be significant unless the saturation is below a particular threshold, which means that traditional PSG parameters, such as the AHI and ODI, are insensitive for predicting the effect of hypoxemia. Nonetheless, further studies are necessary to confirm the role of hypoxemia on perception of sleep quality and identify the possible threshold of hypoxemia in OSA patients.

## Abbreviations

AHI: Apnea-hypopnea index
CPAP: Continuous positive airway pressure
CES-D: Center for Epidemiologic Studies Depression Scale
ESS: Epworth sleepiness scale
HADS: Hospital Anxiety And Depression Scale
OSA: Obstructive sleep apnea
ODI: Oxygen desaturation index
T90: The time spent with saturation < 90 %
PSG: Polysomnography
PSQI: Pittsburgh sleep quality index
SF-36: Short form-36
Saturation-related: parameters
SpO2: Oxyhemoglobin saturation
mean SpO2: Mean saturation
mini SpO2: Minimum saturation
median SpO2: Median value of the SpO2 series
lowest 5 % SpO2: Average value of the lowest five percentage of SpO2 series
highest 5 % SpO2: Average value of the highest five percentage of SpO2 series
Highest 5 % SpO2 minus median SpO2: Difference between the highest 5 % SpO2 and the median SpO2
median SpO2 minus lowest 5 % SpO2: Difference between the median SpO2 and the lowest 5 % SpO2
